# COVID-19 vaccine hesitancy trends in Ghana: a cross-sectional study exploring the roles of political allegiance, misinformation beliefs, and sociodemographic factors

**DOI:** 10.11604/pamj.2022.43.165.37314

**Published:** 2022-12-02

**Authors:** Ken Brackstone, Kirchuffs Atengble, Michael Head, Laud Boateng

**Affiliations:** 1 Clinical Informatics Research Unit, Faculty of Medicine, University of Southampton, Southampton, United Kingdom

**Keywords:** COVID-19, Ghana, West Africa, vaccines, vaccine hesitancy

## Abstract

**Introduction:**

Africa has the slowest COVID-19 vaccination rate of any continent in the world, with only 29.8% of the population receiving at least one dose of the vaccine. This includes Ghana, where only 37.8% of the country have received at least one dose as of October, 2022. The key aims of this research were to determine levels of hesitancy in COVID-19 vaccines among unvaccinated individuals in Ghana and observe their trends across time, and to identify independent predictors associated with vaccine hesitancy among unvaccinated individuals.

**Methods:**

four online cross-sectional surveys of Ghanaian citizens were conducted in August, 2020 (N = 3048), March, 2021 (N = 1558), June, 2021 (N = 1295), and February, 2022 (N = 424).

**Results:**

overall hesitancy decreased from 36.8% (95% CI: 35.1%-38.5%) in August, 2020 to 17.2% (95% CI: 15.3%-19.1%) in March, 2021. However, hesitancy increased to 23.8% (95% CI: 21.5%-26.1%) in June, 2021, and then again to 52.2% (95% CI: 47.4%-57.0%) in February, 2022. Key reasons included not having enough vaccine-related information (50.6%) and concerns over vaccine safety (32.0%). Hesitant groups included Christians, urban dwellers, opposition political party voters, females, individuals who completed higher education, individuals who reported receiving COVID-19 information from internet sources, and individuals who expressed uncertainty about commonly-circulated COVID-19 misinformation beliefs.

**Conclusion:**

hesitancy rates among unvaccinated individuals in Ghana continues to rise. However, vaccine awareness strategies are sensitive to subpopulation characteristics. Many are reachable through targeted communication strategies, to which campaigns must focus on resolving vaccine-related concerns to ensure high vaccine uptake across Ghana.

## Introduction

While high-income settings have achieved relatively high coverage with their COVID-19 vaccination campaigns, 31% of the world´s population are still yet to receive a single dose of any COVID-19 vaccine as of November, 2022 [1]. Africa currently has the slowest vaccination rate of any continent in the world, with only 29.8% of the population receiving at least one dose of the vaccine [1]. This includes Ghana, a country in West Africa with an estimated 30.8 million population. Ghana has reported over 170,000 cases and 1,460 deaths, and currently only 27.9% of the country is considered fully vaccinated with 37.8% receiving at least one dose [2]. With the emergence of the highly-transmissible Omicron variant [3], large-scale vaccination coverage is fundamental to the national and global pandemic response. Three common factors that impact the success of vaccination campaigns include inequity around supply and demand issues, social mobilization and logistical issues (challenges often related to underfunded health programmes), and vaccine confidence once doses arrive in communities. Vaccine hesitancy is defined by the World Health Organization (WHO) as the delay in the acceptance, or blunt refusal of, vaccines. Hesitancy was described by the WHO as one of the top 10 threats to global health in 2019, and has been identified as a growing trend in West Africa more generally [4]. Developing a deeper understanding of the factors associated with vaccine hesitancy is crucial toward informing locally-tailored health promotion strategies.

Vaccine hesitancy in West Africa has been associated with governmental dissatisfaction and mistrust, particularly of government messaging, with a recent example being the boycott of the polio vaccine in Northern Nigeria in 2003-2004 [5]. More recent surveys administered in sub-Saharan African (SSA) countries such as Malawi, Mali, and Nigeria, found that dissatisfaction with the government´s response to the COVID-19 pandemic predicted hesitancy [6]. This pattern has been found in Western countries, including the United States (US) [7], where supporters of opposition political parties (Republicans) demonstrated greater hesitancy to receive the COVID-19 vaccine than supporters of the party currently in government (Democrats). Thus, individual differences in political allegiance may be associated with vaccine hesitancy in Ghana, especially since the most recent Ghanaian election took place in December, 2020 shortly before the first batch of COVID-19 vaccines were delivered to the country.

Studies have also shown that COVID-19-related misinformation is common in West Africa. For example, a nationwide survey conducted in Nigeria found that, whilst respondents were relatively knowledgeable of COVID-19 as a transmissible disease, almost 50% of citizens believed that COVID-19 was a biological weapon designed by the Chinese government, and almost 20% believed that the virus was designed specifically to reduce or control the population [8]. Vaccine misinformation is often amplified on social media [9], especially from specific faith institutions, individuals, and other organized groups that have become deeply entrenched in online platforms such as Facebook [10].

Finally, based on recent research in other African countries [6], vaccine hesitancy is commonly associated with females, richer households, individuals who had completed higher education, and individuals who lived in urban (compared to rural) communities. However, there is still a limited evidence base around COVID-19-related vaccine hesitancy in West Africa and specifically within Ghana. The key aims of this research were to determine levels of hesitancy in COVID-19 vaccines among unvaccinated individuals in Ghana and observe their trends across time, and to identify independent predictors associated with vaccine hesitancy among unvaccinated individuals.

## Methods

**Study design and period:** four cross-sectional surveys were administered. Survey 1 was conducted in August, 2020, three months after the first case of COVID-19 was reported in Ghana and prior to any vaccines being globally approved and available. Survey 2 was conducted in March, 2021 shortly after the first batch of Oxford AstraZeneca (AZ) vaccines arrived in Ghana through the COVID-19 Vaccines Global Access (COVAX) initiative and the beginning of vaccine rollout. Survey 3 was conducted in June, 2021, three months after vaccine rollout begun in Ghana. Finally, survey 4 - intended as a short survey to detect up-to-date hesitancy trends in Ghana - was conducted in February, 2022. Participants completed a self-administered online survey using Qualtrics XM. Each survey was available online for approximately 4 weeks. Dissemination was conducted using a snowball effect of word-of-mouth (WhatsApp, LinkedIn, email, direct messaging), and advertising via Facebook Ads Manager. This technique allowed us to direct the survey toward individuals whose Facebook profile was registered as them being aged 18 and over and residing anywhere in Ghana. Associated study information appeared on individuals´ Facebook timelines along with the survey link. In each survey, participants were offered the opportunity to enter a prize draw to win 1 of 25 money vouchers worth 100 Ghana Cedis (approximately 12 GBP) upon full completion of the survey. Qualtrics allowed checks to ensure that consented participants could only take the survey once from the same internet protocol (IP) address. Informed consent was required to participate in the study after reading the study information statement online. Participants read the study aims and design displayed online, and then checked a tick box to confirm consent.

**Study size determination and sampling technique:** sample size calculations suggested between 385 and 1067 participants in each survey, providing approximately 3-5% margin of error at 95% confidence.

### Outcome variables

**Vaccine hesitancy:** in survey 1, participants were asked: “When the COVID-19 vaccine becomes available to you, would you like to get vaccinated?” (yes, no, I don´t know). In surveys 2-4, participants initially indicated whether they had previously received any doses of the COVID-19 vaccine. Among participants who indicated that they had not received any doses, vaccine hesitancy was then assessed using two distinct measurements. First, participants were asked: “When the COVID-19 vaccine becomes available to you, would you like to get vaccinated?” (yes, no, I don´t know). Participants who indicated disagreement or indecision about receiving the vaccine subsequently specified reasons for their hesitancy. A list of nine reasons was consequently presented (e.g. “(COVID-19) is not serious enough to need a vaccine” [8]). Second, participants indicated the extent of their agreement to the question: “If a vaccine for COVID-19 were available to me, I would get it.” (1 = strongly disagree, 5 = strongly agree).

### Predictor variables

**Others who have received the vaccine:** participants indicated whether they knew anybody personally who had received the COVID-19 vaccine (yes, no) in surveys 2-3.

**Misinformation about COVID-19:** participants indicated whether they believed in seven COVID-19-related misinformation beliefs recorded to be circulating in sub-Saharan Africa by selecting “yes” if they agreed with the belief, “unsure” if they were uncertain about the belief, or “no” if they did not agree with the belief (e.g. “To the best of your knowledge… [COVID-19] is designed to reduce or control the population” [8]). These were assessed in surveys 2-3.

**Sources of COVID-19 vaccine-related information:** participants selected (yes or no) where they typically retrieved COVID-19 vaccine-related information from a list of eight sources [8]. These included common social media platforms (Facebook, WhatsApp, Twitter), more traditional news sources (TV/radio, Ghana Health Service (GHS)), government officials, the internet (e.g. news websites), and family members and friends. These were assessed in surveys 1-3.

**Political allegiance:** participants selected the political party that they voted for in the Ghanaian election of December, 2020 (New Patriotic Party (NPP; elected), National Democratic Congress (NDC; unelected), other, none/I didn´t vote). Participants indicated their trust in the vaccine (*“I would trust the safety of the COVID-19 vaccine when it becomes available to me,”* 1 = strongly disagree, 5 = strongly agree). These were assessed in surveys 2-3.

**Demographic variables:** finally, participants indicated their age, gender, marital status, and religion. Socioeconomic variables included employment status, education, and community type (urban, rural). These were assessed in surveys 1-4.

**Data processing and analysis:** the data captured in Qualtrics were examined for errors, cleaned, and exported into IBM SPSS Statistics 28 for further analysis. Descriptive statistics summarized respondents´ socio-demographics. Inferential statistics were conducted in three phases. First, temporal trends in hesitancy and population prevalence were compared between each survey. For consistency between surveys, hesitancy was coded by dichotomising participants´ responses (no, I don´t know) to the question: “When the COVID-19 vaccine becomes available to you, would you like to get vaccinated?” Chi-Square χ^2^ tests were conducted to assess for categorical differences in hesitancy rates between surveys 1-2, 2-3, and 3-4. Descriptive analyses were also conducted to summarize misinformation beliefs and self-reported sources of vaccine-related information.

In survey 3, bivariate logistic regressions assessed relationships between individual predictors and vaccine hesitancy (S1). A combined logistic regression model was then administered containing all predictors in a single model, providing the strictest test of potential associations with vaccine hesitancy. To account for the level of variance in participants´ responses, vaccine hesitancy was coded by dichotomising participants´ responses (strongly disagree, somewhat disagree, or undecided) to the statement: “If a vaccine for COVID-19 were available to me, I would get it”. Vaccine hesitancy and its associated predictors were rescaled to 0 or 1 in our statistical analyses, which allowed for direct comparison of effect sizes. We focused these analyses on survey 3 as more predictor variables were measured. Finally, a one-way Analysis of variance (ANOVA) and associated post-hoc tests were conducted to compare differences in political groups´ ratings of trust in the COVID-19 vaccine.

**Ethical approvals and consent:** the surveys received ethical approvals from University of Southampton Ethics Committee (Institutional Review Board ID: 57267) and conformed to local ethical standards applied according to the Ghana Health Service (GHS), Ghana.

## Results

**Participants and socio-demographic characteristics:** Table 1 shows descriptive statistics of participants from survey 1 (N = 3048), survey 2 (N = 1558), survey 3 (N = 1295), and survey 4 (N = 424). Among the largest ethnic groups in survey 3 were Akan (46.3%) and Ewe (16.4%). The majority of participants lived in Greater Accra (28.5%) and Ashanti (17.9%) regions, 62.3% had completed higher education, and 37.3% had completed senior secondary education or lower. More than 60% of participants reported being single (62.0%) or married/in a relationship (37.0%), 58.7% reported living in an urban community (compared to rural; 39.0%), and 45.7% reported being unemployed (compared to employed to some degree; 52.0%), while 82.5% reported being Christian (compared to Muslim; 17.5%). Finally, 71.7% reported knowing someone personally who had received the vaccine.

**Table 1:** descriptive statistics of participants from 4 surveys, empty cells indicate variables not collected, note: percentages may not equal 100 due to incomplete questions

Variable	Combined N=6325)	Survey 1 (N=3048)	Survey 2 (N=1558)	Survey 3 (N=1295)	Survey 4 (N=424)
	**% (n)**				
**Gender**					
Male	67.3 (4256)	61.3 (1868)	69.4 (1065)	78.3 (1014)	72.9 (309)
Female	31.8 (2011)	38.1 (1163)	30.0 (467)	20.8 (270)	26.2 (111)
**Age**					
<30	58.7 (3717)	56.1 (1710)	60.7 (945)	64.0 (829)	54.9 (233)
>30	38.1 (2411)	49.3 (1228)	36.7 (572)	32.9 (426)	43.6 (185)
**Marital status**					
Single	63.7 (4032)	69.8 (2129)	56.9 (886)	62.0 (803)	50.6 (214)
Married or in a relationship	35.4 (2237)	30.0 (915)	41.5 (646)	37.0 (479)	46.5 (197)
**Community type**					
Urban	64.1 (2029)		63.7 (992)	58.7 (769)	63.2 (268)
Rural	35.9 (1138)		31.1 (485)	39.0 (512)	33.3 (141)
**Highest education**					
Senior secondary or lower	24.4 (1541)	17.5 (534)	27.3 (426)	37.3 (483)	23.0 (98)
Higher	75.5 (4777)	82.4 (2513)	72.7 (1132)	62.3 (807)	76.7 (325)
**Employment**					
Unemployed	41.3 (2615)	40.2 (1225)	43.0 (670)	45.7 (592)	30.2 (128)
Employed, self-employed, apprentice	58.1 (3676)	59.7 (1821)	56.9 (886)	52.0 (673)	69.8 (296)
**Religion**					
Christian	82.1 (5194)	85.4 (2604)	79.9 (1245)	82.5 (1021)	76.4 (324)
Muslim	14.2 (896)	11.2 (343)	16.6 (258)	17.5 (217)	18.4 (78)
Political allegiance*					
NPP	49.1 (1274)		46.6 (726)	42.3 (548)	
NDC	14.7 (381)		14.0 (218)	12.6 (163)	
Other	8.1 (210)		7.7 (120)	6.9 (90)	
Did not vote	28.1 (729)		24.6 (383)	26.7 (346)	
**Get vaccinated?**					
No	16.3 (1033)	18.5 (563)	9.7 (151)	13.3 (172)	34.7 (147)
I don’t know	13.9 (884)	18.3 (558)	7.4 (116)	10.5 (136)	17.5 (74)
Yes	69.7 (4406)	63.2 (1928)	82.7 (1288)	76.2 (987)	47.8 (203)
**Others vaccine?**					
No	23.0 (395)			28.3 (366)	6.8 (29)
Yes	77.0 (1324)			71.7 (929)	93.2 (395)
**Sources of information**					
Facebook	72.7 (4232)	69.2 (2101)	77.3 (1204)	71.6 (927)	
WhatsApp	54.4 (3715)	84.7 (2570)	49.2 (766)	29.3 (379)	
Twitter	34.5 (2594)	67.3 (2044)	23.9 (372)	13.7 (178)	
Mass media (TV/radio)	75.6 (4367)	68.4 (2078)	85.5 (1332)	73.9 (957)	
GHS or health workers	53.1 (2885)	36.0 (1092)	74.6 (1162)	48.7 (631)	
Government officials	23.6 (1091)	4.1 (121)	41.1 (640)	25.5 (330)	
Family or friends	41.3 (2473)	42.7 (1295)	47.8 (745)	33.4 (433)	
Internet	51.3 (2853)	39.8 (1207)	64.4 (1004)	49.6 (642)	

% (n): results show percentages (%) and number of respondents (in brackets) per question; NPP: New Patriotic Party; NDC: National Democratic Congress; GHS: Ghana Health Service

**Figure 1:**
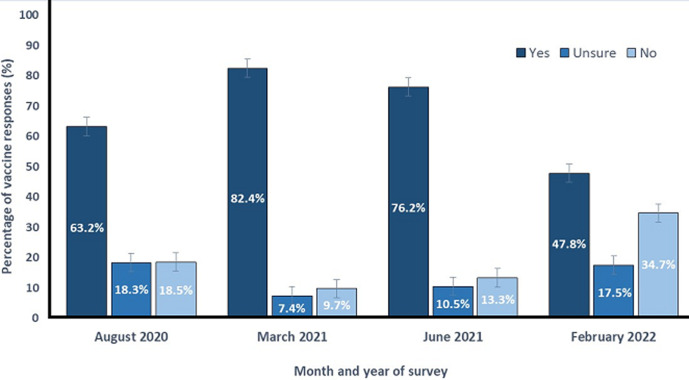
breakdown of yes, no, and unsure responses across four surveys in August, 2020, March, 2021, June, 2021, and February, 2022

**Vaccine hesitancy trends across time:** a Pearson´s Chi-Squared test revealed a significant association (Figure 1) between time and vaccine hesitancy (χ^2^ (1) = 182.687 p < 0.0001), in which overall hesitancy decreased from 36.8% (CI: 35.1%-38.5%) in survey 1 (August, 2020) to 17.2% (95% CI: 15.3%-19.1%) in survey 2 (March, 2021). Hesitancy increased (χ^2^ (1) = 19.188, p < 0.0001) from 17.2% in survey 2 to 23.8% (95% CI: 21.5%-26.1%) in survey 3 (June, 2021). Finally, hesitancy increased again (χ^2^ (1) = 120.413, p < 0.0001) from 23.8% in survey 3 to 52.2% (95% CI: 47.4%-57.0%) in survey 4 (February, 2022).

**Reasons for vaccine hesitancy:** the following analyses derive from survey 3 (June, 2021), which measured more predictors to include in a regression model. Among participants who indicated ‘no´ and ‘I don´t know´ that they would take the vaccine when available (308/1295; 23.8%), the most common reasons were: not having enough information about vaccine (156/308; 50.6%), believing that the vaccine would be unsafe or dangerous (99/308; 32.1%), not trusting the government or service departments (66/308; 21.4%), and believing that they would experience side effects and get sick from the vaccine (58/308; 18.8%; Table 2).

**Table 2:** reasons for refusing the vaccine in March, 2021, June, 2021, and February, 2022, note: empty cells indicate that these variables were not collected

Variable	Survey 2 (N=267/1558)	Survey 3 (N=308/1295)	Survey 4 (N=221/424)
	**% (n)**		
**The vaccine will be dangerous**			
Yes	24.0 (64)	32.1 (99)	47.5 (105)
No	76.0 (203)	67.9 (209)	52.5 (116)
**I will experience side effects and get sick**			
Yes	30.1 (82)	18.8 (58)	33.5 (74)
No	69.3 (185)	81.2 (250)	66.5 (147)
**COVID-19 is not severe enough to need a vaccine**			
Yes	15 (5.6)	14.0 (43)	27.6 (61)
No	252 (94.4)	86.0 (265)	72.4 (160)
**I don’t trust the government department**			
Yes	22.8 (61)	21.4 (66)	16.3 (69)
No	77.2 (206)	78.6 (242)	152 (83.7)
**I will be allergic to the vaccine**			
Yes	9.7 (26)	7.1 (22)	11.8 (26)
No	90.3 (241)	92.9 (286)	88.2 (195)
**It is too far to travel to the vaccination centre**			
Yes	1.9 (5)	6.8 (21)	11.8 (26)
No	98.1 (262)	93.2 (287)	88.2 (195)
**The vaccine will not work**			
Yes	9.7 (25)	10.4 (32)	23.5 (52)
No	90.6 (242)	89.6 (276)	76.5 (169)
**I don’t have enough information about the vaccine**			
Yes		50.6 (156)	52.0 (115)
No		49.4 (152)	48.0 (106)
**I don’t need it**			
Yes		13.0 (40)	19.5 (43)
No		87.0 (268)	80.5 (178)

% (n): results show percentages (%) and number of respondents (in brackets) per question

**Misinformation beliefs:** in all, 44.2% (573/1295) of participants indicated agreement with at least one of the seven widely circulated misinformation beliefs (M = 1.38, SD = 1.97; Table 3). The most common misinformation beliefs included: “… is a biological weapon caused by the Chinese government” (316/1295; 24.4%), “… is a result of 5G technology being installed in Ghana” (310/1295; 23.9%), and “… is a biological weapon designed by the US government” (297/1295; 22.9%). Next, 46.4% (601/1295) of participants indicated uncertainty about at least one COVID-19-related misinformation belief (M = 1.10, SD = 1.55). The most common misinformation beliefs in which participants indicated uncertainty included: “… was designed to reduce or control the population” (347/1295; 26.8%), “… is a biological weapon caused by the Chinese government” (304/1295; 23.5%), and “… is a virus designed by the pharmaceutical industry to sell their drugs” (209/1295; 16.1%).

**Table 3:** breakdown of COVID-19 misinformation beliefs in surveys 2 and 3, note: percentages may not equal 100 due to incomplete questions

Variable	Survey 2 (N=1558)	Survey 3 (N=1295)
	**% (n)**	
**A biological weapon designed by the government of China**		
No	51.0 (794)	48.6 (630)
Yes	16.6 (258)	24.4 (316)
I don’t know	32.4 (506)	23.5 (304)
**A virus designed by the pharmaceutical companies to sell their drugs**		
No	60.1 (936)	58.2 (754)
Yes	10.6 (165)	22.2 (288)
I don’t know	29.3 (457)	16.1 (209)
**An exaggeration by news media to cause fear and panic**		
No	74.6 (1163)	73.2 (948)
Yes	10.2 (159)	9.2 (119)
I don’t know	15.2 (236)	13.7 (177)
**A plague caused by sins and disbelief in human beings**		
No	69.3 (1079)	71.5 (926)
Yes	9.6 (151)	14.3 (185)
I don’t know	21.1 (328)	9.9 (128)
**Designed to reduce or control the population**		
No	54.2 (844)	48.9 (633)
Yes	16.4 (255)	20.8 (270)
I don’t know	29.4 (459)	26.8 (347)
**A biological weapon designed by the United States government**		
No	65.5 (1021)	65.9 (853)
Yes	4.6 (71)	22.9) (297)
I don’t know	29.9 (466)	7.3 (95)
**A result of 5G technology being installed in the country**		
No	61.4 (956)	59.8 (775)
Yes	8.2 (128)	23.9 (310)
I don’t know	30.4 (474)	12.4 (161)

% (n): results show percentages (%) and number of respondents (in brackets) per question

**Sources of COVID-19 vaccine-related information:** the most commonly accessed sources of COVID-19 vaccine-related information were mass media (e.g. newspapers, radio, TV; 73.9% (957/1295)), Facebook (77.3% (927/1295)), the internet (e.g. Google, news websites, blogs; 49.6% (642/1295)), and the GHS (48.7% (631/1295)). Participants were least likely to retrieve vaccine-related information from Twitter (13.7% (137/1295)), government officials (25.5% (330/1295)), WhatsApp (29.3% (379/1295)), and friends or family (33.4% (433/1295)).

**Predictors of vaccine hesitancy:** Table 4 shows the combined logistic regression model of factors contributing to COVID-19 vaccine hesitancy. By political affiliations, participants consisted of NPP voters (elected; 42.3%), NDC voters (unelected; 12.6%), “other” voters (6.9%), and non-voters (26.7%). National Democratic Congress (NDC) voters (opposition) were more likely to be vaccine-hesitant than participants who did not vote (OR: 1.67; 95% CI: 1.07-2.59; p = 0.022). NPP voters were less likely to be vaccine-hesitant than participants who did not vote (OR: 0.57; 95% CI: 0.40-0.80; p = 0.001). A one-way ANOVA showed significant differences in ratings of trust in the COVID-19 vaccine between political groups (F (3,1143) = 16.69, p = 0.0001). Post hoc comparisons using the Tukey honestly significant difference (HSD) test indicated that vaccine-related trust was significantly higher among NPP voters (M = 4.23, SD = 1.60) compared to NDC voters (M = 3.61, SD = 1.39), “other” voters (M = 3.80, SD = 1.35), and non-voters (M = 3.71, SD = 1.35).

**Table 4:** combined logistic regression model of factors contributing to COVID-19 vaccine hesitancy (N = 1138, R^2^ = 0.144)

Variable	OR	p-value	95% CI
Age - older (40+)	1.095	0.591	0.786 - 1.526
Female	1.594	0.008	1.130 - 2.250
Urban community	1.479	0.010	1.099 - 1.989
Married or in a relationship	0.886	0.458	0.644 - 1.219
Higher education (undergrad or postgrad)	1.649	0.002	1.210 - 2.248
Being unemployed	0.903	0.494	0.674 - 1.210
Christian beliefs	2.282	<0.000	1.486 - 3.506
**Political beliefs**			
National Democratic Congress (NDC, unelected)	1.674	0.022	1.074 - 2.597
New Patriotic Party (NPP, elected)	0.575	0.001	0.408 - 0.809
Other political party	0.710	0.249	0.396 - 1.272
Personally know somebody who received vaccine (Y)	0.714	0.041	0.516 - 0.987
Beliefs in misinformation	1.274	0.089	0.864 - 1.683
Uncertainty about misinformation beliefs	1.865	<0.000	1.411 - 2.466
Channels of COVID-19 information			
Facebook	0.907	0.546	0.660 - 1.246
WhatsApp	1.031	0.862	0.728 - 1.461
Twitter	0.956	0.842	0.617 - 1.484
Mass media (e.g. radio, newspapers, TV)	1.367	0.077	0.966 - 1.935
Ghana Health Service or health workers	0.692	0.020	0.508 - 0.943
Government officials	0.734	0.096	0.510 - 1.056
Family members or friends	0.864	0.383	0.623 - 1.200
Internet (e.g. Google, news websites, blogs)	1.388	0.032	1.029 - 1.872

Participants who indicated agreement with at least one misinformation belief (i.e. participants who ticked “yes” to indicate agreement) predicted marginally greater vaccine hesitancy compared to participants who did not indicate misinformation beliefs (OR: 1.27; 95% CI: 0.86-1.68; p =0.089). However, participants who expressed uncertainty in at least one misinformation belief (i.e. those who ticked “I don´t know” to indicate uncertainty) were significantly more likely to express vaccine hesitancy compared to participants who did not indicate uncertainty (OR: 1.86; 95% CI: 1.41-2.46; p < 0.0001). There were no significant predictors of vaccine hesitancy among participants who used social media platforms for COVID-19 vaccine-related information, such as WhatsApp (OR: 1.03; 95% CI: 0.72-1.46; p = 0.862) or Facebook (OR: 0.90; 95% CI: 0.66-1.24; p = 0.546) compared to participants who reported not using these platforms. However, participants who reported using internet webpages (e.g. news websites, blogs) as a source of vaccine-related information were significantly more likely to report hesitancy than those who reported not using the internet (OR: 1.38; 95% CI: 1.02-1.87; p = 0.032). Participants who reported using the GHS as a source of vaccine-related information were less likely to report hesitancy compared to participants who did not report consulting the GHS (OR: 0.69; 95% CI: 0.50-0.94; p = 0.020).

There were several significant demographic and socio-demographic factors. Greater hesitancy was observed in Christian participants compared to Muslim participants (OR: 2.82; 95% CI: 1.48-3.50; p < 0.0001); females compared to males (OR: 1.59; 95% CI: 1.13-2.25; p = 0.008); participants who completed more (compared to less) years of education (OR: 1.64; 95% CI: 1.20-2.24; p = 0.002); participants who lived in urban (compared to rural) communities (OR: 1.47; 95% CI: 1.09-1.98; p = 0.010); and participants who reported knowing somebody (compared to not knowing anybody) who received the vaccine (OR: 0.71; 95% CI: 0.51-0.68; p = 0.041).

## Discussion

This study describes evidence of changes in overall levels of vaccine hesitancy in Ghana across four time-points during the COVID-19 pandemic. Hesitancy decreased between August, 2020 and March, 2021. This occurred around the time of vaccine availability and rollout, and amid extensive health promotion activity. However, hesitancy increased in June, 2021 and continued to increase further in February, 2022. Key reasons for refusing the vaccine included not having enough information about the vaccine and concerns about vaccine safety. Among key groups more likely to express hesitancy included Christians, urban residents, opposition party voters, females, individuals who had completed higher education, individuals who received COVID-19 information from internet sources, and individuals who expressed uncertainty about commonly-circulated COVID-19 misinformation beliefs.

It is possible that the increase in hesitancy rates observed in June, 2021 may have been, in part, due to the global circulation of negative news stories surrounding the Oxford AZ vaccines at the time. The COVID-19 pandemic has been a global news story, and the actions of countries in the global north are seen and absorbed by those in the global south. Hesitancy rates during the pandemic elsewhere in Africa vary greatly, ranging from 2.1% in Ethiopia, 17.3% in Malawi, and 35.5% in Mali [6]. However, our findings are comparable to hesitancy in many higher-income settings, including France [11], South Africa [12], and USA [13]. A pragmatic viewpoint would be that vaccine hesitancy in West Africa, including Ghana, is neither higher nor lower than many other parts of the world.

A perceived lack of information and mistrust were common reasons for hesitancy, along with beliefs in pandemic-related conspiracy theories such as bioweapons, including mistrust in the Ghanaian government. Similar findings were also reported in Nigeria [8] and Malaysia [14]. Voters of the unelected opposition party (NDC) in the Ghanaian general election were more likely to express hesitancy than voters of the elected political party (NPP). Other studies have also demonstrated that political views impact views on vaccination. For example, vaccination rates were significantly lower in counties with a high percentage of US Republican voters [7], and French citizens who indicated voting for a far-right candidate in the previous general election were more likely to state that they would refuse the COVID-19 vaccine if offered [15].

Individuals who positively endorsed misinformation beliefs about COVID-19 contained weak effects on vaccine hesitancy. Thus, it is possible that anxiety caused by endorsing such imaginative beliefs are drivers of both vaccine acceptance and hesitancy. On the other hand, we found that individuals who indicated uncertainty with at least one commonly-circulated misinformation belief about COVID-19 were more likely to express vaccine hesitancy. Perhaps uncertainty associated with one´s beliefs about the COVID-19 pandemic also translates across to beliefs about other man-made developments associated with the pandemic, including vaccines [16]. Nevertheless, these findings should be interpreted with caution. Previous research has shown that the relationship between conspiracy theory beliefs and vaccine acceptance is highly complex, with various psychological dimensions mediating such beliefs and the propensity to get vaccinated, such as death anxiety [17].

Social media use was not a significant predictor of vaccine hesitancy despite WhatsApp and Facebook being two of the most widely used social media platforms in Ghana [18]. However, the use of internet webpages (e.g. news websites, blogs) was associated with hesitancy, suggesting that exposure to websites with potentially sensationalist stories about COVID-19 and vaccines - for example, news outlets that resort to sensationalism and exaggerated superlatives to remain competitive to advertising revenues - could influence individuals´ willingness to receive the vaccine [19]. Further, using the GHS as a source of vaccine-related information was negatively associated with hesitancy, suggesting that information from official health sources may be key to building trust and countering misinformation.

In terms of religion, Christians were more hesitant than Muslims. A small number of churches have promoted anti-vaccine viewpoints, including the Christ Embassy, with headquarters in Nigeria and multiple churches in Ghana [20,21]. Previously in northern Nigeria, religious leaders developed misconceived perceptions about the polio vaccine. Many communities view religious leaders as a trustworthy and credible source of health advice and information, with research showing that religious leaders´ opinions can strongly influence social and behavioural norms [22]. However, for some religious individuals - particularly among those with strong beliefs of a controlling god - scientific inventions work against their core beliefs about the world [23], and may highlight potential difficulties of persuading some religious leaders to promote pro-vaccination messaging to their followers. Given that the majority of participants in this study were Christians, it is not surprising that a large number of participants of Christian faith expressed doubts about vaccination. However, without in-depth examination, it is difficult to conclude whether Christianity is a true predictor in this study, or if it is the strength of conviction that they held about their religion.

Urban residents were more likely to be hesitant than rural residents, also reflected in research from Burkina Faso, Ethiopia, and Malawi [6]. Urban residents are typically more connected to the internet and social media and thus may be more exposed to vaccine-related misinformation than rural inhabitants who have fewer sources of information available to them. Thus, urban residents may be more worried about side effects and more likely to avoid vaccination, or are less afraid about COVID-19 due to having more access to information and daily alerts about COVID-19 [24,25].

Finally, females were more likely to be hesitant than males, similar to findings from Nigeria [6], and participants with more years of education were more likely to be hesitant than less educated participants, perhaps reflecting how more educated people in our sample are less likely to conform to social norms and behaviours [22]. This compares with surveys conducted in Burkina Faso, Ethiopia, Malawi, and Nigeria [6]. However, the opposite is true in research from Malaysia [14] and Uganda [26].

The core strength of this study relates to its relatively large number of participants, including temporal comparisons across four surveys. However, the need for internet access limits the representativeness of the sample population. Thus, certain demographic was under-represented, including individuals in rural areas and people of lower socio-economic status. Since our recruitment was also conducted using cross-sectional convenience sampling methods, there will also be presence of respondent bias. But the efficiency of data collection, the lower cost to advertise, and the acceptability of online survey recruitment may provide a useful alternative than formal regional or national surveys, especially during a global pandemic where new information from population surveys via remote or virtual methods may be urgently required. We also completed the same survey via in-person data collection in a rural area of the Oti Region in January, 2022 [27].

## Conclusion

Hesitancy rates among unvaccinated individuals in Ghana continue to rise. Hesitancy decreased between August, 2020 and March, 2021. However, hesitancy increased in June, 2021 and continued to increase further in February, 2022. Key reasons for refusing the vaccine included not having enough information about the vaccine and concerns about vaccine safety. Among key groups more likely to express hesitancy included Christians, urban residents, opposition party voters, females, individuals who had completed higher education, individuals who received COVID-19 information from internet sources, and individuals who expressed uncertainty about their COVID-19 misinformation beliefs. Health promotion campaigns should use locally and nationally trusted knowledge providers (e.g. the GHS) and disseminate good public health messaging via local trusted individuals. For instance, messages should be distributed through religious groups or on media platforms that are utilized by hesitant population groups. Many of these groups are reachable through targeted communication strategies, to which campaigns can focus on resolving concerns about vaccine-related side effects, and provide reassurance about the safety of approved COVID-19 vaccines to ensure high uptake and low vaccine hesitancy across Ghana.

**Funding:** this study was funded by the Global Challenges Research Fund (GCRF) Strategic Development Fund, University of Southampton, and the Economic and Social Research Council (ESRC) Impact Acceleration Account (IAA). The funders had no interference with the conduction, analysis, and publication process.

### What is known about this topic

- *The vast majority of unvaccinated people reside in lower-income settings in sub-Saharan Africa (SSA), including Ghana; Ghana has reported over 167,000 cases and 1456 deaths, and currently only 26.5% of the country is considered fully vaccinated;*
- *Vaccine hesitancy - defined as the delay in the acceptance, or blunt refusal of, vaccines, is one hindrance to the success of vaccination campaigns;*
- *There is limited up-to-date evidence on vaccine hesitancy trends in Ghana, including reasons and independent predictors for COVID-19-related vaccine hesitancy among unvaccinated individuals*.

### What this study adds

- *Vaccine hesitancy decreased from 36.8% in August, 2020 to 17.2% in March 2021, however, hesitancy increased to 23.8% in June, 2021, and then again to 52.2% in February, 2022;*
- *Key reasons included not having enough vaccine-related information and concerns over vaccine safety; new groups associated with vaccine hesitancy included opposition political party voters, individuals who received COVID-19 information from internet sources, and individuals who expressed uncertainty about commonly-circulated COVID-19 misinformation beliefs*.
